# Older adults and family caregivers’ experience of digital health technology in frailty care: A systematic review and meta-ethnography protocol

**DOI:** 10.12688/hrbopenres.13549.1

**Published:** 2022-05-13

**Authors:** Andrew Darley, Rachael Dix, Elena Rocher, Diarmuid Stokes, Áine Carroll

**Affiliations:** 1 School of Medicine, University College Dublin, Dublin, Ireland; 2 Las Naves, Valencia, Spain; 3 Health Sciences Library, University College Dublin, Dublin, Ireland; 4 The National Rehabilitation Hospital (NRH), Dublin, Ireland

**Keywords:** digital health, eHealth, ageing, frailty, meta-ethnography

## Abstract

**Background:** Digital health technology has been identified as a valuable tool to support older adults with frailty needs in their home setting. Despite the numerous technologies and evaluations of these innovations, a synthesis of the older person and family caregivers’ experience using technology for support self-management has not been conducted to date.

**Methods and analysis:** A systematic review and meta-ethnography will be conducted in accordance with the PRISMA and eMERGe reporting guidelines. Four peer-reviewed empirical evidence databases will be searched (Medline (Ovid), CINAHL, EMBASE, PsycINFO) using a defined search strategy. Studies containing qualitative data on the experiences of older people or family caregivers of using digital health technology to support frailty care will be included. Covidence software will be used to screen studies and extract data. The Critical Appraisal Skills Programme (CASP) checklist for qualitative research will be used by two independent reviewers to appraise all included papers. A meta-ethnography will be undertaken in accordance with the seven-phase method described by Noblit and Hare: (1) Getting started, (2) Deciding what is relevant to the initial interest, (3) Reading the studies, (4) Determining how the studies are related, (5) Translating the studies into one another, (6) Synthesizing translations and (7) Expressing the synthesis.

**Discussion:** To the best of our knowledge, this will be the first systematic review to integrate and synthesize the findings of qualitative studies of older citizens’ experience of digital health technology. The findings of this meta-ethnography will endeavour to inform future research, policy and clinical practice. In particular, the results will help to inform the design of future digital health technology to meet the needs of older adults.

PROSPERO registration number: Submitted 05/04/2022 and currently under review.

## Introduction

Healthy and active ageing have become a global health priority as life expectancy is increasingly improving ^1^. It is estimated that by 2050, the median age of several European Union (EU) countries will have risen by up by eight years ^2^. However, this increase in an individual’s lifespan does not infer better quality of life. With longer lifespans, people are at a higher risk of experiencing a chronic health condition which, in turn, places greater demands on the management of health and social care services ^3^. Coupled with the increasing lifespans of our population are the decreasing fertility rates, which in turn have led to rising old-age dependency ratios across the EU. It is estimated that by 2050, the average of this ratio will be 50%, meaning that there will be less than two people of working age for every person over the age of 65 ^2^, creating an unsustainable social security system that will not be sufficient to support the care of older people. 

European government expenditures are increasingly burdened to cover the costs of chronic health conditions such as frailty, heart disease, cancer, diabetes, stroke, and arthritis ^4^. It has been reported that these conditions account for 70–80% of public health expenditure in the EU, which amounts to an estimated €700 billion ^5^. In the EU in 2018, approximately 37% of adults over the age of 65 were reported to have two or more chronic conditions ^6^. The presence of chronic health conditions may limit one's capacity to carry out the activities of daily living (ADL), such as personal hygiene or grooming, dressing, toileting, transferring or ambulating, and eating, or instrumental activities of daily living (IADL), such as transportation and shopping, managing finances, shopping and meal preparation, house-cleaning and home maintenance, managing communication with others, medication management ^7^. The inability to perform ADLs and IADLs implies further pressure on government expenditure, as long-term care assistance may be required ^8^. In addition to government spending, out-of-pocket payments for individuals with chronic health conditions are higher due to increased health-care use, lower earnings due to disability, early retirement, and dependence on social security systems ^9– 11^.

The burden of care on family or informal caregivers is an additional factor to consider in relation to chronic conditions, the predominant profile being female and between the ages of 45 and 64 years ^12^, and accounts for 75% of the care provided to older dependent people ^13^. Informal caregiving may create physical and psychological strain over extended periods of time and has the capacity to create secondary stress across multiple life domains such as work and family relationships ^14^; these caregivers have also been referred to as ‘invisible second patients’ when caring for older adults ^15^.

A common health condition amongst older people, often experienced with other chronic comorbidities, is frailty. Frailty is a distinct health condition related to the ageing process whereby multiple physiological systems gradually lose their intrinsic capacity which can result in sudden health status changes due to stress or an event such as a fall or an infection ^16^. The prevalence of frailty increases with age, ranging between 4 and 59% in populations of home-dwelling older people and is higher in women than in men ^17^. People living with frailty are known to have more falls, more disabilities, use more medications and require more access to long-term healthcare services than individuals without the condition ^18^. Frailty is not inevitable and can be avoided, delayed, and reversed with timely and appropriate interventions such as self-management and care planning ^19^. It has been estimated that intervening against frailty would reduce the rates of dependency in older people by up to 40–50%. This reduction in dependency would imply an improvement in the quality of life of millions of European citizens and would significantly decrease European public spending ^20^. Therefore, older adults must be encouraged to engage in proactive health behaviours that prevent or support individuals in maintaining their intrinsic capacity as they age.

Digital health has been identified as a valuable method in supporting older adults’ self-management and well-being in their home setting ^21, 22^. As a concept, no singular definition of digital health exists. In their recently published Global Strategy for Digital Health 2020–2025, The World Health Organisation (WHO) ^23^ referred to it as “the field of knowledge and practice associated with the development and use of digital technologies to improve health”, which will be utilised in the current protocol. Christophorou and colleagues ^24^ identified several contexts in which digital health can promote active and healthy aging such as prolonging time in the working environment, overcoming social isolation and loneliness, accessing public and private services, and stimulating independence. Digital health technology enables healthcare providers to engage, motivate and promote healthy lifestyle behaviours amongst older adults, and includes but is not limited to, smartphone/tablet applications, websites, connected devices, video consultations and wearable tracking devices ^25^. A substantial light was shone on digital technology and its value in supporting older people during the coronavirus disease 2019 (COVID-19) pandemic, when many older adults were required to stay in their homes, restricting their social contacts and preventing them from visiting their usual care centres, due to being considered a high-risk population ^26^.

While available empirical reviews detail several types of digital health interventions for older people with various conditions ^27– 34^, there is a lack of uniformity between the condition included, intervention type and methods for evaluation in terms of their usability, acceptability and efficacy ^35^. More specifically, digital health interventions focusing on frailty care amongst older adults are scarce and rarely assess their effectiveness. This is compounded by the lack of agreement regarding the condition of what ‘frailty’ refers to. Many of the studies to date have failed to scientifically evaluate the benefits of digital health interventions in terms of frailty reversal and quality of life ^35^. Indeed, authors of a systematic review on technology for ageing in place reported a lack of outcome measurements in the studies included in their analysis ^36^. One scoping review on digital health interventions among people living with frailty reported validated assessment outcomes in only 45% of the studies included ^35^. In a systematic review performed by Kampmeijer *et al.* ^25^, the authors outlined the evidence on the facilitating factors and barriers to the use of digital health tools for health promotion and primary prevention among older adults. However, evidence on the effectiveness of the tools was not discussed in the review.

While it is evident that there has been increasing interest in the development and assessment of technologies for older people with frailty, to the best of the authors’ knowledge, there are currently no evidence synthesis regarding which digital health interventions better support older citizens living with frailty in their home setting. Therefore, it is difficult to determine if digital health can effectively promote independent living and/or ageing in place for older people with frailty, and if so, which types of digital health interventions are more effective. Moreover, there has been no effort to synthesise family caregivers’ experience of digital health interventions for frailty, whether they directly used the technology or supported a relative who did.

In a recent scoping review by Linn and colleagues ^35^ the main objective was to provide a broad overview of digital health interventions used for people living with frailty, to identify gaps in the literature, and to describe the robustness of the digital approaches. The authors of this review highlighted the heterogeneity among frailty assessments, study designs and evaluations of digital health interventions within the included studies, making it difficult to draw conclusions on their effectiveness. Furthermore, it was found that few studies evaluated efficacy, usability or feasibility and that frailty assessment of their sample was commonly not reported in over a third of the included studies ^35^. In addition, for the studies that did assess and report their sample’s frailty levels, lack of uniformity existed regarding the frailty assessment employed and frailty scores of participants. It should also be considered that assessment of the caregivers’ experience with the digital health intervention was not discussed, highlighting another area that requires further investigation. Linn and colleagues ^35^ concluded their review with a call for “ *standardized approaches to assess frailty, well-structured randomized controlled trials, and proper evaluation and report*”.

Taking the conclusions of Linn and colleagues’ ^35^ review into consideration, it is evident that there is a need for a standardized approach to investigating the area of digital health interventions in frailty care. Acknowledging that reviews in the area suggest that the effectiveness of such interventions remains encouraging yet ambiguous, it is important to broaden the scope of the evaluation by examining the experiences of older people and family caregivers using technology, rather than simply measuring their effectiveness rates or health outcome changes. While usability and efficacy are central to evaluation of digital health to ensure they achieve their intended purpose, it may also prove insightful to understand how older people and their family caregivers responded to using such technology and what it personally meant for them in their daily lives. In order to effectively contribute to the current body of knowledge, the authors of the original review were consulted in the development of the current protocol. It was determined that an in-depth qualitative synthesis was necessary regarding the evidence to date, which is the approach set forth in the current protocol.

### Research aims

The aims of the current protocol are (a) to systematically search empirical qualitative literature to identify studies exploring the experiences of older adults and family caregivers using digital health to support the management of frailty in the home setting, and (b) to perform a meta-ethnography to synthesise the included studies with the intention of describing the phenomenon and identifying new insights. The following research questions were established, based on the SPIDER tool ^37^, which will guide the review and the qualitative synthesis of identified literature:

1. What are older adults and their family caregivers’ experiences of digital health technology to support frailty care in the home setting?
2. What facilitators and barriers exist to using digital health technology for frailty care in the home setting amongst older adults and their family caregivers?

## Protocol

The systematic review protocol is written in accordance with The Preferred Reporting Items for Systematic Review and Meta-Analysis Protocols (PRISMA-P) statement which is publicly available ^38^. Qualitative studies identified in the review will be subject to a meta-ethnographic study, which is an inductive, highly interpretive approach ^39^. A meta-ethnographic approach has been chosen as the method of qualitative evidence synthesis for this review because it “provides the opportunity for us to carefully consider the relationship between studies, understand the issues and to comprehend the reality of everyday life” ^39^. Meta-ethnography was chosen for its ability to explore existing qualitative data and formulate comprehensive interpretations or theoretical frameworks using existing evidence. Meta-ethnography has been previously used in healthcare settings to examine care transitions of older people and their caregivers ^40^, physical activity in older age ^41 ^and digital technology ^42^.

Meta-ethnography will be used to synthesize and evaluate the results of the included studies in accordance with Noblit and Hare’s ^39^ seven stages: (1) Getting started, (2) Deciding what is relevant to the initial interest, (3) Reading the studies, (4) Determining how the studies are related, (5) Translating the studies into one another, (6) Synthesizing translations and (7) Expressing the synthesis. These steps will be explained in further detail below in how they relate to the current protocol. The review will be reported in line with the eMERGe guidance for reporting meta-ethnography ^43^. Ethical approval is not required for the synthesis of published peer-reviewed studies and their related data.

### Phase 1: Selecting meta-ethnography and getting started

Phase one of a meta-ethnography involves reporting the rationale and the context for the study. To the best of our knowledge, no meta-ethnography exists to date which synthesises the evidence on the experiences of older adults with frailty using digital health technology in their home setting. Following the authors’ discovery of the comprehensive scoping review by Linn and colleagues ^35^, a gap in the knowledge existed regarding a deeper and focused examination of qualitative evidence on this topic. A key aim of the review and rationale for utilising meta-ethnography is to provide a conceptual understanding of how older adults and their family caregivers experience and engage with digital health technology within the context of frailty care.

### Phase 2: Deciding what is relevant to research aim

***Search strategy.*** The Sample, Phenomenon of Interest, Design, Evaluation, and Research type (SPIDER) tool ^37^ was used to structure the search terms. A search strategy ( Table 1) was developed based on the research questions, with key terms from this strategy tested in a scoping search of the literature. Relevant keywords and phrases will be used in each database. Keywords and phrases include older adults, family caregivers, digital health technology and experiences. The search strategy was reviewed by a university research librarian for comprehensiveness. The search strategy will be adapted and conducted within four empirical databases: Medline (Ovid), CINAHL, EMBASE and PsycINFO. Search results will be limited to articles written in English dated from 2010 to the present day. Reference lists of relevant articles will also be manually searched by the author team to identify further studies.

***Study selection.*** The SPIDER search strategy tool ^37^ also informed the eligibility criteria to identify the relevant literature. Table 2 outlines each aspect of the SPIDER tool (sample, phenomenon of interest, design, evaluation, and Research Type) and its related inclusion/exclusion criteria. Included studies will describe a sample of older adults (65+) with a diagnosis of mild or moderate frailty and their family or informal caregivers. Given the lack of definition internationally regarding the medical condition of frailty, where adults do not have a formal diagnosis but identify as having frailty needs will still be included in the screening process. 

**Table 1.** Search terms for systematic review.

	Search terms
**Sample**	“older pe*” OR “older adult*” OR “older citizen” OR “senior*” OR elder* OR “ageing population*” OR “ageing adult*” OR “old age*” OR “older age” OR “geriatric*” OR “older individual*” OR elder* OR “aged” **AND** frail* OR “challenges in the activities of daily living” OR ADLs OR pre-frail **AND** Family care* OR caregiver* OR family OR informal care* OR carer* OR spouse* OR partner OR friend* OR relative* OR guardian* OR dyad*
**Phenomenon of** **interest**	“digital health*” OR “digital intervention*” eHealth OR e-Health OR telemedicine OR “tele-medicine” OR telecare OR tele-care OR “electronic health” OR “internet health” OR telehealth OR tele-health OR “mobile health” OR “mHealth” OR m-Health OR “mobile app*” OR “tablet app*” OR “Medicine 2.0" OR teleconsultation*
**Design**	Interview* OR “focus group*” OR “co-design” OR “participatory research*”
**Evaluation**	experience OR patient-perspective* OR accept* OR feasibility OR usability OR expectation OR understanding OR comprehension OR perception* OR attitude*
**Research type**	qualitative OR “mixed method*”

**Table 2.** Sample, Phenomenon of Interest, Design, Evaluation, and Research type tool (SPIDER) table of study inclusion and exclusion criteria.

	Inclusion	Exclusion
**Sample**	• Older citizens, aged ≥65+ living with frailty in their home setting • Family caregivers to older citizens (≥65+) living with frailty in the home setting	• Individuals under the age of 65 with frailty • Older people with significant cognitive impairment • People with multiple conditions where frailty is not the primary focus • People living in institutional settings ( *e.g.*, hospital or nursing home, age-friendly supported housing)
**Phenomenon of** **interest**	• Experience of digital health in their home setting	• Experience of digital health in care setting • Experience of digital health where frailty is not the main condition • Studies on perception or attitudes with no direct experience reported • Digital health design studies regarding how they could be supported
**Design**	• Qualitative or mixed-method studies reporting primary qualitative data collected using qualitative methods (through direct observation; focus groups or interviews)	• Studies that report quantitative data only including questionnaire studies with open- ended free text questions
**Evaluation**	• Qualitative analysis of lived experience of using digital health technology including feasibility, acceptability, facilitators, barriers	• Studies that evaluate using quantitative methods only • Studies that do not explicitly state the method of analysis
**Research type**	• Peer-reviewed journal articles using qualitative design or including a qualitative component that are distinguished from other methods used • Full text available in English language	• Reviews; protocols; theoretical work; editorials; opinion pieces and grey literature • Non-English language

When the search has been conducted in each database, the identified articles will be imported into the bibliographic reference manager, Mendeley, to remove any duplicates before initial screening by reviewers. The citations will then be exported to the screening and data extraction software tool Covidence, to screen all articles using the eligibility criteria. Each article will be required to be approved by two independent reviewers (AD, RD) before either being included or excluded in the review. A pilot testing of articles (n=10) using the Covidence software package and inclusion and exclusion criteria will be undertaken by the authors to ensure consistency of the methodology adopted in the selection process ^44^. Full-text screening will then be carried out on all articles that meet the inclusion criteria during the initial screening round by two independent reviewers (AD, RD). In the instance of disagreement regarding an article’s inclusion, a third reviewer (AC) will additionally independently assess the relevance of the article and decide the final outcome. A PRISMA flow diagram will be created once all screening has been completed to ensure transparency of the process.

***Quality appraisal.*** Despite the lack of consensus regarding quality appraisal for qualitative research, the authors will include this step in the evidence identification for the purpose of critically evaluating the evidence and the rigor and transparency. The methodological quality of the included studies will be appraised using the ten-item Critical Appraisal Skills Programme (CASP) checklist for qualitative research. The CASP tool is widely used in qualitative research and has been recommended for use in health research. Two evaluators (AD, RD) will independently assess the quality of each study with disagreement resolved through consensus and discussion with a third evaluator (ER), if necessary. No studies will be excluded based on quality; however, the appraisal will be used to highlight methodological limitations in their interpretation of the study findings, particularly when developing their own synthesis.

### Phase 3: Reading the studies

This stage of the meta-ethnography will involve repeated careful reading and immersion in the identified studies to obtain familiarity, and to determine the key concepts and author interpretations relevant to the study aims. Following repeated close reading of the identified articles, the raw data for the meta-ethnographic synthesis will be extracted into a collaborative data extraction form in a Microsoft Word document between the study authors. First-order constructs ( *i.e.*, participant quotations) and second-order constructs ( *i.e.*, author metaphorical themes or concepts) will be extracted from each article at this point ^45^. A separate data extraction form will also be created ^46^ to describe study contextual information, such as study setting, participants, research design and aim, frailty measured and description of the digital health technology. Throughout this phase, two reviewers will initially complete coding and data extraction independently, before working together to discuss discrepancies in their analysis and identify emerging themes.

### Phase 4: Determining how the studies are related

This phase will involve determining the relationships between the key concepts from the range of papers. In this context, a ‘concept’ refers to a “meaningful idea that develops by comparing particular instances” where they must explain and not just describe the data ^43^. In order to understand the relationship between concepts amongst the various studies, the authors will create a list of themes to conduct a close comparison and assess the common and recurring concepts between studies. From this list, the themes from the studies will be clustered into relevant categories, in which common concepts will be grouped according to similar underlying metaphors ^46^. These categories will be labelled using terms that encompass the key concepts they contain. This phase will be iterative whereby revisions may be conducted based on author discussions and the source text.

### Phase 5: Translating the studies into one another

This phase involves a constant comparison of the studies to examine the key concepts within. The translations will be conducted in chronological order ^47^. Each concept from each paper will be compared with all other articles in turn to reciprocally assess the presence or absence of the concept, with the aim of organising the concepts in further conceptual categories ^48^. Team discussions regarding the key concepts and their meanings will be held to ensure rigor and challenge interpretations of the data. The two lead authors (AD, RD) will maintain two independent journals during this phase to ensure the transparency of analysis, and that they are aware of their own theoretical position ^49^.

### Phase 6: Synthesizing the translations

During this phase, third-order constructs ( *i.e.*, the reviewer’s higher-order interpretations of first- and second-order constructs) will be established with the intention of “making the whole into something more than the parts alone imply” ^50^. The authors will view the studies as a whole, rather than individual studies, to garner a conceptual framework ^45, 48^ which will explain the phenomenon of interest. This phase will be conducted in two steps. In the first step, a reciprocal and refutational synthesis will be performed, in which the authors will determine the similarities and dissimilarities between the studies. This step will help inform whether the synthesis will focus their cohesion (reciprocal translation synthesis), contradictions (refutational synthesis) or whether both are necessary ^46^. A line of argument synthesis will then be created from third-order constructs by the lead authors independently (AD, RD) through constant comparison to create higher-order interpretations using the identified internal concepts within the identified studies. Once this step is carried out independently, the two lead authors will merge their findings to produce the final line of argument synthesis and will be reviewed by the wider team.

### Phase 7: Expressing the synthesis

The authors will follow the eMERGE reporting guidelines ^43^ when writing up the synthesis for dissemination. Meta-ethnographic reporting will focus on the (i) summary of findings (ii) strengths, limitations and reflexivity and (iii) recommendations and conclusions ^43^. Findings from the meta-ethnography will be published in a peer-reviewed journal, presented at relevant international conferences and made available to the general public and patients, in a suitable format. When creating material for the general public, including older people, we will work with an older person advocacy organisation to ensure that the media are acceptable and accessible to the population and disseminated using the most effective and engaging channels.

## Conclusions

A cornerstone in the management and treatment of frailty is health behavioural change and lifestyle in the home setting, such as diet and exercise, to maintain one’s intrinsic capacity. As such, digital health technology has been valued for its potential to promote independent and autonomous living in older people. To the best of our knowledge, this will be the first systematic review to integrate and synthesize the findings of qualitative studies of older adults with frailty and their experience of digital health. This systematic review and meta-ethnography protocol describes the method in which to address vital gap in the existing literature on lived experiences of older adults using digital health technology. We believe that a focus on how older adults have experienced self-management technology in their home setting is essential to understanding how to expand and make progression in the field. While more research is being invested in deciding how to collaborate and support this population, it is important to take stock of the evidence to date in what is known about their lived experience to help shape what it is to come. Without this understanding, researchers and technology inventors may be destined to repeat previous approaches that were not acceptable or effective amongst this population. While the results of the review may not be generalisable to all older adults, we believe it is important to focus on the specific health condition of frailty to understand the nuances and needs of those who experience it. The findings of this review are intended to inform future research and policy, digital technology design and clinical practice.

## Data availability

### Underlying data

No data are associated with this article.

### Reporting guidelines

Figshare: PRISMA-P Checklist for “Older adults and family caregivers’ experience of digital health technology in frailty care: A systematic review and meta-ethnography protocol”, https://doi.org/10.6084/m9.figshare.19657053.v1 ^38^

Data are available under the terms of the Creative Commons Attribution 4.0 International license (CC-BY 4.0).
