# Supplementary material for: Older adults and family caregivers’ experience of digital health technology in frailty care: A systematic review and meta-ethnography protocol
Source: HRB Open Res. 2022 Aug 18;5:38. Originally published 2022 May 13. [Version 2] doi: 10.12688/hrbopenres.13549.2 (PMC9391740; doi:10.12688/hrbopenres.13549.2)
Supplement: Peer review history

# Peer review: Reviewer response for version 2

**DOI:** 10.21956/hrbopenres.14872.r32703

**Published:** 2022-08-19

**Authors:** Áine Teahan

**Affiliations:** 1 Trinity College Dublin, Dublin, Ireland

Many thanks to the author for addressing and replying to my comments. This is an important area of research particularly in the context of ageing, family carers and digital health. I have no further comments and look forward to reading future findings.

Is the study design appropriate for the research question?

Yes

Is the rationale for, and objectives of, the study clearly described?

Partly

Are sufficient details of the methods provided to allow replication by others?

Yes

Are the datasets clearly presented in a useable and accessible format?

Not applicable

Reviewer Expertise:

I am currently working on a research project exploring digital health for older people with multi-morbidity. My previous research has focused on psychosocial support for family carers of people with dementia. I am broadly familiar with the field of frailty. I have also conducted systematic reviews in the past.

I confirm that I have read this submission and believe that I have an appropriate level of expertise to confirm that it is of an acceptable scientific standard.

# Peer review: Reviewer response for version 1

**DOI:** 10.21956/hrbopenres.14791.r32114

**Published:** 2022-06-06

**Authors:** Gloria A. Aguayo

**Affiliations:** 1 Department of Precision Health, Luxembourg Institute of Health, Strassen, Luxembourg

This document is a protocol of a systematic review and meta-ethnography about the experiences of older adults with digital health technology. The protocol is well-written. The research question is relevant for the older population with frailty but not only for this population. The introduction gives a comprehensive background for the study citing the most important literature in the field. It has an adequate methodology with details describing a standardised stud selection using the SPIDER search strategy tool and the seven steps for meta-ethnography and its standardised guideline (eMERGe).

Is the study design appropriate for the research question?

Yes

Is the rationale for, and objectives of, the study clearly described?

Yes

Are sufficient details of the methods provided to allow replication by others?

Yes

Are the datasets clearly presented in a useable and accessible format?

Not applicable

Reviewer Expertise:

Healthy ageing, frailty, diabetes.

I confirm that I have read this submission and believe that I have an appropriate level of expertise to confirm that it is of an acceptable scientific standard.

# Peer review: Reviewer response for version 1

**DOI:** 10.21956/hrbopenres.14791.r32112

**Published:** 2022-06-01

**Authors:** Áine Teahan

**Affiliations:** 1 Trinity College Dublin, Dublin, Ireland

Thank you for the opportunity to review this paper. This is a well-written protocol for a systematic review and meta-ethnography for older people with frailty and family carers. I look forward to reading future findings.

 I have made some suggestions and raised some points for clarification below.

**Introduction:**

 The figure 4-59% prevalence of frailty in older people is quite a big range. It would be helpful if the authors could reference the weighted prevalence of 10.7% reported as well (Collard *et al.*, 2012). ^1^ Also the weighted prevalence of pre-frailty (41.6%) would provide additional background on the importance of research in this area.

 If available, it would be useful if the authors could reference some examples of meta-ethnographic approaches applied to digital health and older people with other health conditions. This would provide some concrete examples of the benefits of this methodology in digital health.

**Research Aims:**

 I am unclear on the first aim- does this systematic review aim to explore the experiences of older adults and family carers (i.e. care dyads)? Or does the SR aim to explore the experiences of older adults and/or family carers?

 If the latter, the sample search terms in Table 1 will need slight modification. As they’re currently designed this search strategy require that both older people with frailty and family carer key words need to be present. This could miss literature focusing on older people but not family carers and vice versa.

**Methods:**

 Depending on the authors’ response to my research aims query- Table 1 may require an edit.

 For replicability, it would be useful if the authors’ would specify within what limits each string will search i.e. title, abstract, full-text etc- Table 1.

 The authors identified four peer-reviewed databases- is peer review an inclusion/exclusion criteria for the review? Will the hand-searched citations need to be peer reviewed too?

Is the study design appropriate for the research question?

Yes

Is the rationale for, and objectives of, the study clearly described?

Partly

Are sufficient details of the methods provided to allow replication by others?

Yes

Are the datasets clearly presented in a useable and accessible format?

Not applicable

Reviewer Expertise:

I am currently working on a research project exploring digital health for older people with multi-morbidity. My previous research has focused on psychosocial support for family carers of people with dementia. I am broadly familiar with the field of frailty. I have also conducted systematic reviews in the past.

I confirm that I have read this submission and believe that I have an appropriate level of expertise to confirm that it is of an acceptable scientific standard, however I have significant reservations, as outlined above.

## References

[1] : Prevalence of frailty in community-dwelling older persons: a systematic review. *J Am Geriatr Soc* .2012;60(8) : 10.1111/j.1532-5415.2012.04054.x 1487-92 10.1111/j.1532-5415.2012.04054.x 22881367

# Response

**Published:** 2022-08-12

**Authors:** Andrew Darley

**Affiliations:** University College Dublin, Ireland

Dear Reviewers,

 We are grateful for the time and effort committed by you in evaluating our manuscript. Based on your feedback, we have amended the paper and provided responses to each comment below. We believe we have appropriately addressed your suggestions and the protocol is stronger as a result.

 Best Wishes,

 The Author Team  

**Reviewer Comment**

**Introduction: **The figure 4-59% prevalence of frailty in older people is quite a big range. It would be helpful if the authors could reference the weighted prevalence of 10.7% reported as well (Collard *et al.*, 2012). ^1^ Also the weighted prevalence of pre-frailty (41.6%) would provide additional background on the importance of research in this area.

**Author Response**

 Thank you for this suggestion and statistics. We have reworded this statement to include the weighted prevalence for frailty and pre-frailty.

 If available, it would be useful if the authors could reference some examples of meta-ethnographic approaches applied to digital health and older people with other health conditions. This would provide some concrete examples of the benefits of this methodology in digital health.

**Author Response**

 We have identified two studies that adopted meta-ethnography to study older adults’ experience of digital health. We were unable to identify studies specific to frailty care. This has been included under ‘Protocol’ where the method is explained and refers to other healthcare settings in which it has been applied.

**Research Aims:**

 I am unclear on the first aim- does this systematic review aim to explore the experiences of older adults and family carers (i.e. care dyads)? Or does the SR aim to explore the experiences of older adults and/or family carers?

 If the latter, the sample search terms in Table 1 will need slight modification. As they’re currently designed this search strategy require that both older people with frailty and family carer key words need to be present. This could miss literature focusing on older people but not family carers and vice versa.

**Author Response**

 The review will focus on exploring experiences of older adults and/or family carers. We have amended the search strategy to clarify and reflect the target sample

**Methods:** Depending on the authors’ response to my research aims query- Table 1 may require an edit.

**Author Response**

 This has been edited based on previous comment.

 For replicability, it would be useful if the authors’ would specify within what limits each string will search i.e. title, abstract, full-text etc- Table 1.

**Author Response**

 The search strategy in each database will be limited to Title and Abstract. We have included a statement this specification under ‘Search Strategy’.

 The authors identified four peer-reviewed databases- is peer review an inclusion/exclusion criteria for the review? Will the hand-searched citations need to be peer reviewed too?

**Author Response**

 The review will focus on evidence that has been peer reviewed and is a criteria to be included. We will be searching CINAHL which contains both peer-review and grey literature so would only include the peer-reviewed material. We have included a sentence under ‘Search Strategy’ to explicitly state this.
